# Molecular analysis of the AGXT gene in Syrian patients suspected with primary hyperoxaluria type 1

**DOI:** 10.1186/s12920-021-00996-x

**Published:** 2021-06-03

**Authors:** Hossam Murad, Mohamad Baseel Alhalabi, Amir Dabboul, Nour Alfakseh, Mohamad Sayah Nweder, Youssef Zghib, Hala Wannous

**Affiliations:** 1 Human Genetics Division, Molecular Biology and Biotechnology Department, Human Genetics Division, Atomic Energy Commission of Syria, P.O. Box 6091, Damascus, Syria; 2 Chlidien’s Hospital of Damascus, Damascus, Syria

**Keywords:** Primary hyperoxaluria type 1, AGXT, Syria

## Abstract

**Background:**

Characterization of the molecular basis of primary hyperoxaluria type 1 (PH-1) in Syria has been accomplished through the analysis of 90 unrelated chromosomes from 45 Syrians patients with PH-1 from different regions.

**Methods:**

Alanine glyoxylate aminotransferase (AGXT) gene mutations have been analyzed by using molecular detection methods based on the direct DNA sequencing for all exons of the AGXT gene.

**Results:**

Seventeen pathogenic mutations were detected in our patients. Six mutations were novels. The three most frequent mutations were c.33_34insC (p.Lys12fs) in Exon 1, c.584 T < G; p.Met195Arg in exon 5 and c.1007 T > A (p.Val336Asp) in exon 10, with a frequency of 33.3%, 12.2%, and 11.1%, respectively.

**Conclusion:**

DNA sequencing used in this study can offer a useful method to investigate the mutations in Syrian PH-1 patients, and could offer an accurate tool for prenatal diagnosis and genetic counseling.

## Background

Primary hyperoxaluria (PH) is a recessive inherited inborn error of glyoxylate metabolism. The estimated prevalence of PH is 1–3 per 1,000,000 individuals [1]. Formerly, three subgroups are well known within PH. Primary hyperoxaluria type 1 (PH-1; MIM 259900) is an autosomal recessive and inherited disorder. It considered as the most severe form of PH. It is caused by mutations in alanine glyoxylate aminotransferase (AGXT) gene encode the alanine: glyoxylate aminotransferase (AGT) enzyme (AGT; EC2.6.1.44) [2].

The absence of the enzyme AGT leads to the conversion of glyoxylate to oxalates, and thus causes an increase in blood oxalate levels (hyperoxalemia) as well as urine oxalate levels (hyperoxaluria) with large deposits of calcium oxalate in the kidneys and other organs [3]. While, the deficiency of glyoxylate and hydroxypyruvate reductase, which is encoded by GRHPR, is responsible for primary hyperoxaluria type 2 (PH-2). Primary hyperoxaluria type 3 (PH-3) is caused by mutations in HOGA1. This gene encodes the mitochondrial enzyme, 4-hydroxy-2-oxoglutarate aldolase (HOGA) which catalyzes the 4-hydroxy-2-oxoglutarate to glyoxylate and pyruvate [4, 5].

Patients with PH-1 have variable clinical features at presentation, like, urinary tract infections abdominal pain, recurrent hematuria, nephrolithiasis, nephrocalcinosis and end-stage renal disease (ESRD) [5]. Age at onset of symptoms typically varies from 1 to 25 years. About, 20–50% of patients have advanced chronic kidney disease (CKD) or even end stage renal disease (ESRD) at the time of diagnosis [6, 7].

AGXT present in two haplotypes: Major allele (Maj) and Minor allele (Min). The minor allele have three polymorphisms: Proline to leucineat residue 11 (P11L) in exon 1, Isoleucine to Methionine at residue 340 (I340M) in exon 10 and 74 bp duplication in intron 1. Major allele is present about 100% in the peroxisome, while the minor allele is present about 95% in the peroxisome and 5% in the mitochondria [8]. The polymorphic variants are found in linkage disequilibrium with the minor allele haplotype, whereas the absence of these polymorphisms defines the major allele.

AGXT gene is located on chromosome 2 (2q37.3) and spans about 10 kb DNA. The protein of this gene consists of 392-amino-acid (43 kDa). Mutations in the AGXT gene moderated the PH-1 phenotype. To date, there are more than 200 mutations that have been described as causing the disease according to the Human Gene Mutation Database (HGMD) (http://www.hgmd.cf.ac.uk). Seventy-five percent of these mutations are classified as point mutations (nonsense, missense, and nucleotide changes affecting splice-site consensus sequences). While, 25% of these mutations are detected as minor or major deletions and insertions [6].

In this study, we performed sequence analyses of AGXT gene to characterize the mutational spectrum of PH-1 in Syrian patients.

## Methods

### Patients

A total of 45 unrelated patients with PH-1 deficiency from different regions from Syria, corresponding to 90 independent alleles, were enrolled in this study. The average age of patients at referral was 7.18 years, ranging between 3 months and 45 years. Clinical manifestations of the patients with PH was based on (nephrocalcinosis, urolithiasis and end-stage renal failure), urinalysis (raised oxalate) and elevated plasma oxalate in combination with spectrophotometric analysis of the calculation (Table 1).

**Table 1:** Features of patients with mutation

#	Sex	Symptoms onset age Y/M	Allele 1	Allele 2	Initial compliant	Consanguinity	Familial renal stone history	Urine Oxalate (mg/1.73 m2/24 h)
F1	F	15 years	c.584 T > G (p.Met195Arg)	c.584 T > G (p.Met195Arg)	Trf	Yes	Yes	70
F4	F	13 years	c.33_34insC (p.Lys12fs)	c.33_34insC (p.Lys12fs)	Trf	Yes	No	55
F6	F	8 years	c.33_34insC (p.Lys12fs)	c.33_34insC (p.Lys12fs)	Ruti + Rf	Yes	No	82
F8	F	4 years	c.602A > T (p. Asp201Val)	c.602A > T (p. Asp201Val)	Aki	Yes	No	55
F9	F	8 years	c.1007 T > A (p.Val336Asp)	c.1007 T > A (p.Val336Asp)	Aki + Bks	Yes	No	45
F10	F	3 years	c.941C > T (p.Pro314Leu)	N	Ruti & Oli	No	No	50
F19	F	13 years	c.33_34insC (p.Lys12fs)	c.33_34insC (p.Lys12fs)	Ruti + Rf + Hem	No	Yes	55
F21	M	1 y	c.322 T > C (p.Trp108Arg)	c.322 T > C (p.Trp108Arg)	Rf	Yes	No	80
F23	M	3.5 years	c.33_34insC (p.Lys12fs)	c.33_34insC (p.Lys12fs)	Ruti + Ssp	Yes	Yes	282
F25	M	7 months	c.971-972delTG (p.Val324fs)	c.971-972delTG (p.Val324fs)	Rf	Yes	No	250
F28	M	3 months	c.33_34insC (p.Lys12fs)	c.1007 T > A (p.Lys12fs)	An	Yes	Yes	150
F30	F	8 months	c.33_34insC (p.Lys12fs)	c.33_34insC (p.Lys12fs)	Rf	Yes	No	240
F31	F	7 months	c.33_34insC (p.Lys12fs)	c.33_34insC (p.Lys12fs)	Bneph	Yes	No	128
F33	F	8 years	c.305 T > A (p.Val102Asp)	c.305 T > A (p.Val102Asp)	Ruti	Yes	Yes	92
F34	F	2.5 years	c.584 T > G (p.Met195Arg)	c.584 T > G (p.Met195Arg)	Ruti	Yes	Yes	60
F35	F	7.5 months	c.198C > A (p.Tyr66X)	c.198C > A (p.Tyr66X)	Trf + An	Yes	Yes	50
F36	F	7 months	c.866G > A (p.Arg289His)	c.866G > A (p.Arg289His)	Trf	Yes	No	30
F39	F	10 years	c.322 T > C (p.Trp108Arg)	c.322 T > C (p.Trp108Arg)	Rf	Yes	No	75
F40	F	8 years	c.508G > A (p.Gly170Arg)	c.997A > T (Arg333X)	Trf	No	No	200
F41	F	10 months	c.1007 T > A (p.Val336Asp)	c.1007 T > A (p.Val336Asp)	Ruti	Yes	No	130
F42	M	3.5 years	c.584 T > G (p.Met195Arg)	c.1078C > T (p.Arg360Trp)	Trf	No	Yes	250
F45	F	5 months	c.33_34insC (p.Lys12fs)	c.508G > A (p.Gly170Arg)	Aki	Yes	Yes	230
F48	M	1 months	c.971-972delTG (p.Val324fs)	c.971-972delTG (p.Val324fs)	Aki	Yes	Yes	111
F49	M	12 years	c.33_34insC (p.Lys12fs)	c.33_34insC (p.Lys12fs)	Rf	Yes	No	150
F50	M	4 months	c.33_34insC (p.Lys12fs)	c.33_34insC (p.Lys12fs)	Aki	Yes	Yes	100
F51	F	2.5 years	c.584 T > G (p.Met195Arg)	c.584 T > G (p.Met195Arg)	Ruti + Rf	Yes	Yes	368
F56	F	6 years	c.359-1_382del	c.359-1_382del	Bks + Rf	yes	yes	294
F60	F	2 years	c.33_34insC (p.Lys12fs)	c.508G > A (p.Gly170Arg)	Bks + Ruti	No	No	150
F61	M	5 months	c.322 T > C (p.Trp108Arg)	c.322 T > C (p.Trp108Arg)	Rf	Yes	Yes	107
F65	M	3 months	c.33_34insC (p.Lys12fs)	c.1007 T > A (p.Val336Asp)	Aki	No	No	90
F66	F	8 years	c.584 T > G (p.Met195Arg)	c.584 T > G (p.Met195Arg)	Bks + Ruti	Yes	Yes	297
F68	F	4 months	c.33_34insC (p.Lys12fs)	c.508G > A (p.Gly170Arg)	Aki	No	Yes	75
F73	M	3 years	c.33_34insC (p.Lys12fs)	N	Bks	No	No	202
F86	M	4 y	c.603C > A (p.Asp201Glu)	c.603C > A (p.Asp201Glu)	Bks + Hem	Yes	Yes	100
F93	M	9 years	c.584 T > G (p.Met195Arg)	c.584 T > G (p.Met195Arg)	Trf	Yes	No	130
F99	M	8 months	c.33_34insC (p.Lys12fs)	c.33_34insC (p.Lys12fs)	Bneph	Yes	Yes	200
F100	F	7 years	c.33_34insC (p.Lys12fs)	c.33_34insC (p.Lys12fs)	Bneph	Yes	No	150
F103	M	7 years	c.1007 T > A (p.Val336Asp)	c.1007 T > A (p.Val336Asp)	Ruti	Yes	No	150
F111	M	13 years	c.140G > A (p.Gly47Glu)	c.731 T > C (p.Ile244Thr)	Bks	Yes	Yes	150
F116	F	45 years	c.1007 T > A (p.Val336Asp)	c.1007 T > A (p.Val336Asp)	Bks	Yes	Yes	125
F123	M	5 years	c.198C > A (p.Tyr66X)	c.198C > A (p.Tyr66X)	Nakt	Yes	Yes	80
F133	M	5 years	c.198C > A (p.Tyr66X)	N	ND	No	Yes	47
F134	M	4.5 years	c.971-972delTG (p.Val324fs)	c.971-972delTG (p.Val324fs)	Aki	Yes	Yes	160
F136	M	4 years	c.33_34insC (p.Lys12fs)	c.33_34insC (p.Lys12fs)	Rf	Yes	No	125
F138	F	9 years	c.33_34insC (p.Lys12fs)	c.33_34insC (p.Lys12fs)	Bneph	Yes	Yes	246

#	Blood creatinine	Calcemia (mg/dl)	Urine oxalate	Urine creatinine	Radiological findings	Additional information	ESRD	Outcome
F1	13	7	High	34	Bneph	Hemdia	Yes	Died
F4	11	7.2	High	21	Bneph	Perdia	Yes	ND
F6	2	8	High	11	UL	Sr	No	Alive
F8	3.7	6.6	High	13	Bneph	ND	Yes	ND
F9	10	9.9	High	10	UL	ND	Yes	Died
F10	0.4	8	High	18	UL	ND	?	ND
F19	5.6	6	High	16.5	UL	ND	Yes	Alive
F21	1.5	7.7	High	12	Bneph	ND	No	ND
F23	0.6	8.4	High	35	UL + Bneph	Cm	No	Alive
F25	9	9.6	High	43	Bneph	Perdia	Yes	Died
F28	7	8.5	High	12	Bneph	ND	Yes	Died
F30	8.9	7.3	High	23	Bneph	ND	Yes	Died
F31	0.82	9.7	High	13	Bneph	Cm	No	Alive
F33	0.6	9.2	High	8	UL	Cm	No	Alive
F34	0.76	10	High	11	Bneph	ND	No	ND
F35	8.3	9.2	High	15	Bneph	Perdia	Yes	Died
F36	6.7	7	Normal	10	UL + Bneph	Perdia	Yes	ND
F39	1	5	High	15	Bneph	Perdia	No	ND
F40	10	8	High	10	Bneph	Perdia	No	ND
F41	0.7	12	High	19	Bneph	Hemdia	No	ND
F42	2.5	11	High	22	UL	Perdia	No	ND
F45	9	9.5	High	15	Bneph	Hemdia	Yes	ND
F48	4.3	8.5	High	10	Bneph	Hemdia	Yes	Died
F49	5	8	High	12	Bneph	Cm	Yes	Alive
F50	14	8.5	High	15	Bneph	ND	Yes	Died
F51	5.3	7.9	High	24	Bneph	Perdia	Yes	Died
F56	3.5	7	High	10	Bneph	Cm	Yes	Alive
F60	0.6	10	High	6.5	Bneph	Perdia	NO	Alive
F61	4	9	High	15	Bneph	Cm	Yes	Died
F65	8.9	8	High	20	Bneph	Cm	Yes	ND
F66	1.4	9	High	20	UL	Hemdia	No	Alive
F68	3.5	7.8	High	16	Bneph	ND	Yes	Died
F73	0.38	9.5	High	20	UL	Perdia	No	Alive
F86	0.7	10	High	22	UL	Perdia	No	Alive
F93	9.6	8.7	High	15	Bneph	Hemdia	Yes	Died
F99	0.8	8.5	High	15	Bneph	Perdia	No	Alive
F100	0.9	9	High	24	Bneph	Perdia	No	ND
F103	0.9	9	High	24	Bneph	Cm	No	Alive
F111	4	9.5	High	25	Bneph	Hemdia	Yes	Alive
F116	4	8	High	15	Bneph	Drakt	Yes	Alive
F123	1.7	10	High	67	Bneph	Cm	Yes	Alive
F133	3.7	8	Normal	44	Bneph	ND	Yes	Alive
F134	0.74	8.8	High	30	Bneph	Cm	no	Alive
F136	1.4	9.1	High	15	Bneph	Cm	No	Alive
F138	1	10.6	High	28	Bneph	ND	Yes	ND

*Trf* terminal renal failure; *An* Anuria, *Ruti* recurrent urinary tract infection, *Rf* renal failure, *Aki* acute kidney injury, *Bks* bilateral kidney stones, *Oli* oliguria, *Hem* hematuria, *Ssp* spontaneous stone passage, *Nakt* nephrocalcinosis after kidney transplantation, *Hemdia* hemodialysis, *Perdia* peritoneal dialysis, *Sr* stone resection, *Cm* conservative management, *Drakt* disease recurrence after kidney transplant, *Bneph* bilateral nephrocalcinosis, *UL* urolithiasis, *ESRD* end stage renal disease, *Min* miner, *Maj* major, *ND* no data aviable

### DNA isolation and sequencing

Blood samples (3 ml) were collected from each patient by venipuncture in EDTA. Genomic DNA was isolated from peripheral blood using the QIAamp DNA Blood Mini Kit (Qiagen, Inc.) according to manufacturer's instructions. All exons and flanking regions of the AGXT gene were amplified by PCR and screened for mutations by direct sequencing as previously reported [9]. Obtained sequences were aligned to the AGXT reference genomic sequence (GenBank: NM_000030.2).

### Ethical approval

The parents of the minor patient and all adult patients, provided informed consent to the diagnostic and therapeutic procedures involved, in agreement with the guidelines approved by our institutional clinical research ethics committee.

## Result

### Clinical data of PH-1 patients

Our study was composed of 45 unrelated PH-1 patients from different regions of Syria: twenty-five patients (55.55%) were female and twenty (44.44%) were male. The parents of 75% of these patients had blood relation (positive consanguinity). Twenty-three of 45 patients (51.1%) had a positive family history of renal stone (Table 1).

### Identification of mutations

Molecular diagnosis was performed at first by DNA direct sequencing of exons 1, 2, 5, 7 and 10 in order to investigate the common mutations reported in Arab population [10–13]. In heterozygous and negative patients for tested exons, we performed a molecular analysis of the remaining exons of AGXT gene.

**Fig. 1:**
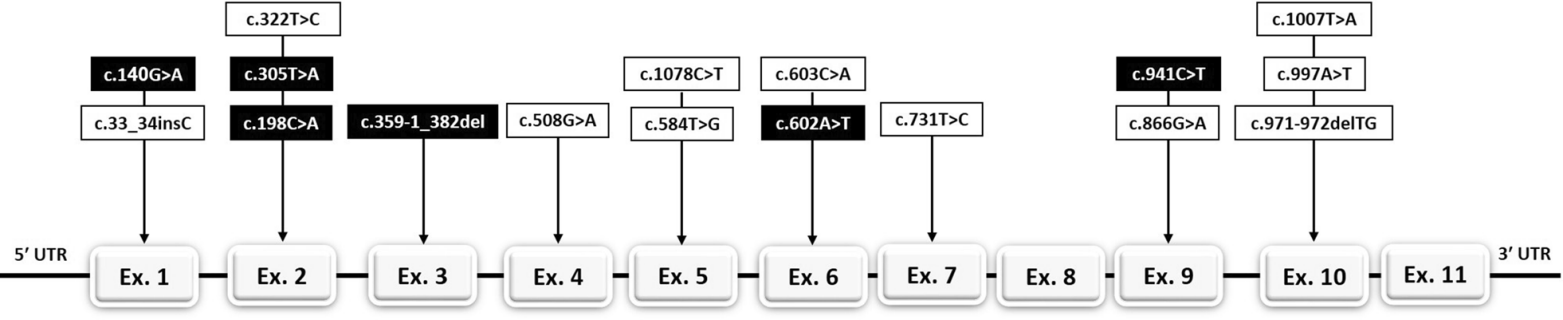
Schematic representation of the AGXT gene with the localization of the mutations found in this study

Analysis of the coding regions and flanking intronic sequences of the AGXT gene shows the presence of seventeen different mutations in 45 patients (Fig. 1).

Thrifty-four patients (75.5%) had disease causing homozygous mutations; eight patients (17.7%) had disease causing compound heterozygous mutations. Three additional individuals were found to be carriers of the one mutation (Table 1).

The most frequent mutation carried by our patients was the c.33_34insC (p.Lys12fs) in exon 1 with an allele frequency equal to 33.3%. The second most frequent mutation was T to G substitution at position 584 (c.584 T < G; p.Met195Arg) with allele frequency, 12.2%. The mutations p.Val336Asp, p.Trp108Arg and p.Val324fs showed 11.1%, 6.7% and 6.7% respectively of the allele frequency. These five mutations therefore account for approximately 70% of the mutant alleles in this gene (Table 2).

**Table 2:** Results of molecular analysis for DNA from 45 PH-1 Syrian patients

Mutation	Exon	Maj/min	Number of alleles	Allele frequency (%)
c.33_34insC	1	Maj	30	33.3
c.584 T > G	5	Min	11	12.2
c.1007 T > A	10	Min	10	11.1
c.322 T > C	2	Min	6	6.7
c.971-972delTG	10	Maj	6	6.7
c.198C > A	2	Maj	5	5.6
c.508G > A	4	Min	4	4.4
c.305 T > A	2	Min	2	2.2
c.602A > T	6	Min	2	2.2
c.866G > A	9	Maj	2	2.2
c.603C > A	6	Maj	2	2.2
c.359-1_382del	3	Maj	2	2.2
c.731 T > C	7	Min	1	1.1
c.140G > A	1	Min	1	1.1
c.941C > T	9	Min	1	1.1
c.1078C > T	11	Maj	1	1.1
c.997A > T	10	Maj	1	1.1

**Fig. 2:**
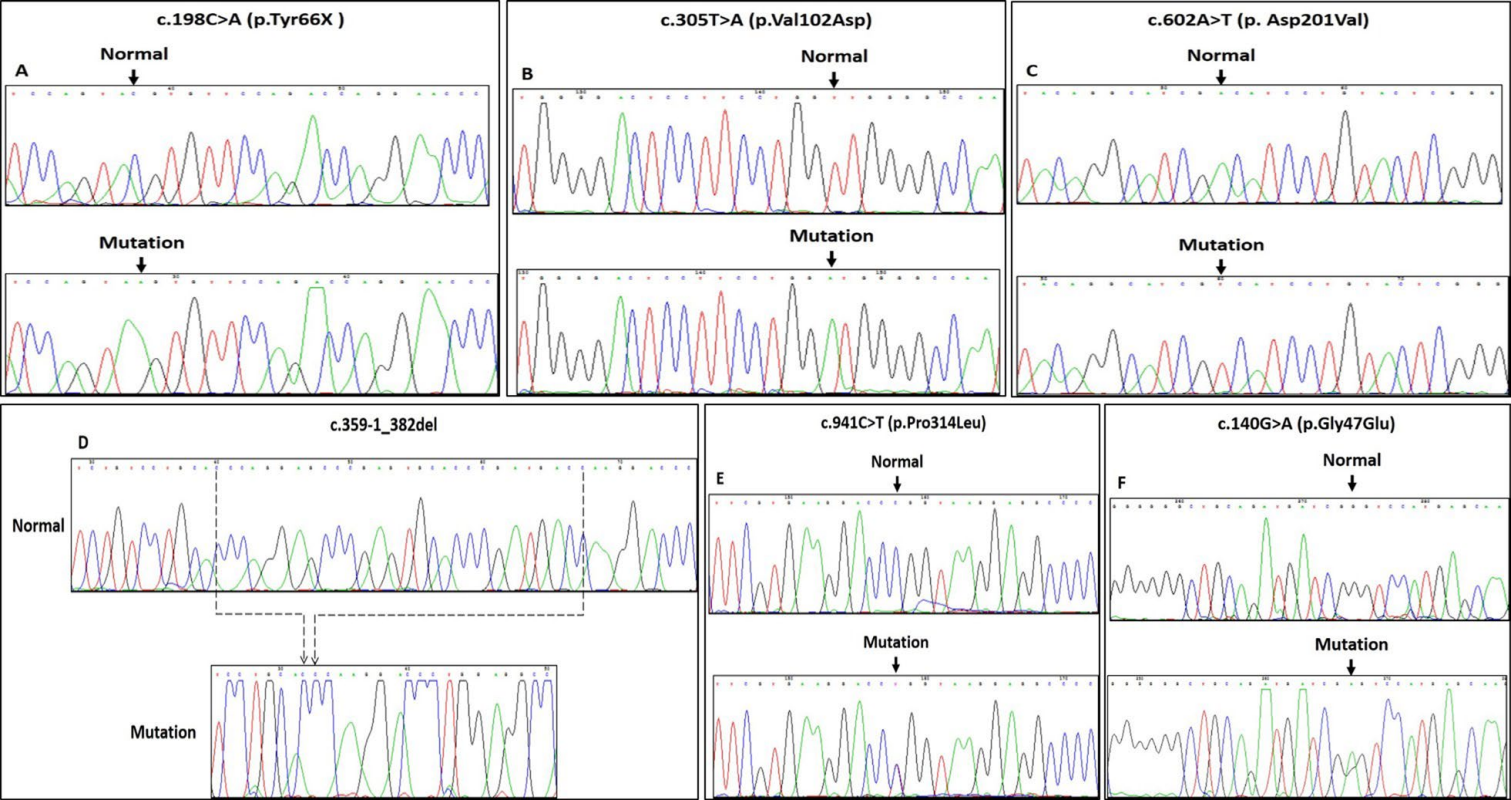
**a**–**f** Sequence chromatograms of the c.198C > A (p.Tyr66X), c.305 T > A (p.Val102Asp), c.602A > T (p. Asp201Val), c.359-1_382del, c.941C > T (p.Pro314Leu) and c.140G > A (p.Gly47Glu) mutations in the AGXT gene. Arrow shows the sequence change

On the other hand, c.140G > A (p.Gly47Glu), c.198C > A (p.Tyr66X), c.305 T > A (p.Val102Asp), c.602A > T (p. Asp201Val), c.941C > T (p.Pro314Leu) and c.359-1_382del mutations were detected as novel mutations (Fig. 2a–f). The first one was nonsense mutation resulting in a truncated protein, and the other four were missense mutations, while the sixth one was a small deletion (del 83 bp). The two mutations c.198C > A (p.Tyr66X), and c.602A > T (p. Asp201Val) were previously reported in Human Gene Mutation Database (HGMD) (http://www.hgmd.cf.ac.uk) with other variations for the same cDNA position. The rest of mutations have not been previously described in the HGMD or the PH Mutation Database [14]. Mutations of patients are summarized in Tables 1.

### Polymorphisms and haplotype analysis

Haplotype analysis performed based on sequencing data for 11 SNPs (Table 3). The analysis showed that some mutations segregate with a specific genotypic combination. Minor allele (p.P11L, p.I340M and 74-bp duplication in intron1) and the three polymorphisms c.165 + 16A > G (rs66494441) in intron 1, p.Ala88Ala (rs35698882) in Exon 2, and c.358 + 13C > T (rs34995778) in intron 2 co-segregated with the four mutations p.Val102Asp, p.Trp108Arg, p.Val336Asp and p.Met195Arg. Additional variations, p.Asn22Ser (rs34885252), c.423 + 29C > T (rs117043148) and the c.*41C > A (rs4273214) were associated with the p. Asp201Val mutation.

**Table 3:** Polymorphism and haplotype analysis detected PH 1 patients

Mutation	Ex. 1		I.1		Ex. 2	I.2	I.3	I.5	Ex. 6	Ex. 10	3,	Haplotype
	c.32C > T	c.65A > G	c.165 + 16A > G	c.165 + 19_165 + 92dup74	c.264C > T	c.358 + 13C > T	c.423 + 29C > T	c.595 + 100G > A	c.654G > A	c.1020A > G	c.*41C > A	
	p.Pro11Leu	p.Asn22Ser			p.Ala88Ala				p.Ser218Ser	p.Ile340Met		
	rs34116584	rs34885252	rs66494441	rs180177174	rs35698882	rs34995778	rs117043148	rs12997245	rs33958047	rs4426527	rs4273214	
c.305 T > A (p.Val102Asp)	TT	AA	GG	+	TT	TT	CC	GG	GG	GG	CC	Min
c.322 T > C (p.Trp108Arg)	TT	AA	GG	+	TT	TT	CC	GG	GG	GG	CC	Min
c.1007 T > A (p.Val336Asp)	TT	AA	GG	+	TT	TT	CC	GG	GG	GG	CC	Min
c.584 T > G (p.Met195Arg)	TT	AA	GG	+	TT	TT	CC	GG	GG	GG	CC	Min
c.602A > T (p. Asp201Val)	CC	GG	GG	+	CC	CC	TT	GG	GG	GG	AA	Min
c.359-1_382del	CC	AA	AA	–	CC	CC	CC	GG	GG	AA	CC	Maj
c.33_34insC (p.Lys12fs)	CC	AA	AA	–	CC	CC	CC	GG	GG	AA	CC	Maj
c.603C > A (p.Asp201Glu)	CC	AA	AA	–	CC	CC	CC	GG	AA	AA	CC	Maj
c.866G > A (p.Arg289His)	CC	AA	AA	–	CC	CC	CC	AA	GG	AA	AA	Maj
c.971-972delTG (p.Val324fs)	CC	AA	AA	–	CC	CC	CC	AA	GG	AA	CC	Maj
c.198C > A (p.Tyr66X)	CC	AA	AA	–	CC	CC	CC	AA	GG	AA	CC	Maj

On the other hand, the c.359-1_382del and p.Lys12fs mutations co-segregated with the major allele. We noted also that, intronic polymorphisms c.595 + 100G > A (rs12997245) in introns 5 is linked with the p.Arg289His, p.Val324fs and p.Tyr66X mutations. In addition, the polymorphism p.Ser218Ser (rs33958047) in exon 6 linked with the p.Asp201Glu mutation. Furthermore, the c. ∗ 41C > A in coding region 3’UTR was associated with the p. Asp201Val and p.Arg289His mutations. These results revealed the founder nature of the reported mutations.

## Discussion

PH-1 is a rare, recessive, inherited inborn error of glyoxylate metabolism. It remains underdiagnosed because of the large variability in its clinical presentation and age of onset. For that, all patients with recurrent nephrocalcinosis or nephrolithiasis should be evaluated for PH-1 [15].

High rates of consanguinity in Middle East countries had a clear effect in increasing the frequency of patients with PH-1[10, 12, 13, 15]**.**

This is the first study about the mutational screening for PH-1 among Syrian children. In our 45 patients, the diagnosis of PH-1 was initially based on clinical findings, elevated plasma oxalate, urine analysis (raised oxalate), and spectrophotometric analysis of the calculation urine showing a pure calcium oxalate monohydrate (whewellite).

Previous studies reported that mutations found in exons 1, 2, 5, 7 and 10 are responsible for 70% of PH-1 patients in Arab population [10, 12, 13]. Therefore, these five exons were firstly screened by DNA sequencing.

This approach allowed us to identify genetic basis in 37 patients (37/45; 82.22%), the remaining patients (8/45; 17.77%), we tested exons 3, 4, 6, 8, 9 and 11 of the AGXT gene.

In our study, among the 45 patients in Syria, we found 17 different mutations in the AGXT gene that cause PH-1. This result confirms the high degree of genetic heterogeneity in this disease. Six of these mutations (c.140G > A, c.198C > A, c.305 T > A, c.602A > T, c.941C > T and c.359-1_382del) have not been previously reported. The vast majority of the patients were homozygous for their mutations, as could be expected in a highly consanguineous community. Identification of molecular bases in all investigated PH-1 patients allowed us to propose a mutational screening strategy for the Syrian population.

c.33_34insC (p.Lys12fs) was the most frequently detected mutation in our study. c.33_34insC is a mutation that generates a stop codon, which leads to the formation of a truncated protein [16]. This duplication is spread in many ethnic and geographic regions in the world, and its high frequency could probably be attributed to multiple mutations in the region of eight cytosine repeat sequences where it occurs.

The second most common mutation found was the c.584 T > G (p.Met195Arg) mutation (12.2%), which is a mutation in a well conserved region of exon 5. This sequence change replaces methionine with arginine at codon 195 of the AGXT protein (p.Met195Arg). Some individuals affected with primary hyperoxaluria, type 1 had this variant [17–19]. This variant has been described to affect AGXT protein function [20]. Prediction software estimated that this variant may create or strengthen a splice site, but this estimation has not been confirmed. For these causes, the c.584 T > G variant has been known as Pathogenic.

The c.1007 T > A (p.Val336Asp) is the third most common mutation in our cohort (11.1%). Most patients affected by this mutation were in a homozygous condition, only two were heterozygous. This mutation results in the amino acid replacement Val336Asp. The mutation was present on the minor allele and in combination with the frequent c.33_34insC (p.Lys12fs). The Val336Asp mutation appears to affect in a negative way with the effect of pyridoxine, and in fact, prompts the development of renal insufficiency, at least in homozygous patients [7].

The c.322 T > C (p.Trp108Arg) mutation was found in 6.7% of our cohort. It was identified in a homozygous state in our patients. This sequence change replaces tryptophan with arginine at codon 108 of the AGXT protein (p.Trp108Arg). This variant is observed in many individuals affected with AGXT-related conditions [17, 21]. This variant has been known to affect AGXT protein function [20, 22, 23]. For these causes, this variant has been considered as Pathogenic.

In addition, the c.971-972delTG (p.Val324fs) mutation was reported with 6.7% of allele frequency in three patients. It was identified in a homozygous state. It was associated with the Maj allele. This variation was reported without exact frequencies or origin of patients [6].

The AGXT c.508G > A (p.Gly170Arg) variant (4.4%) is previously well defined as one of the most common variants associated with primary hyperoxaluria type 1 [9, 24, 25]. It was identified in our cohort in a compound heterozygous state with two different mutations c.997A > T (Arg333X), and c.33_34insC (p.Lys12fs), and no patients were reported to be homozygous for this mutation. Functional studies confirm that the G170R variant is linked with mistargeting of the AGT enzyme to the mitochondria more than to the peroxisomes [26]. On the other hand, several studies informed that, patients with G170R mutation may have longer preservation of renal function with conventional treatment compared to other pathogenic mutations and respond to pyridoxine treatment which is a cofactor that reduces enzyme mistargeting [27–29].

The c.866G > A (p.Arg289His) and c.603C > A (p.Asp201Glu) mutations were found in a homozygous state in 2.2% of cases. The two mutations co-segregate with the Min allele.

The c.866G > A variant has been reported as a part of two complex alleles in patients with Primary Hyperoxaluria Type 1 [6, 30]. On the other hand, the variant c.603C > A (p.Asp201Glu) has been detected in many individuals and families affected with hyperoxaluria [9, 27, 30]. This variant causes a replacement of aspartic acid residue by glutamic acid residue at codon 201 of the AGXT protein (p.Asp201Glu). This variant has been known to affect AGXT protein function [20]. For these reasons, this variant has been predicted as Pathogenic.

The mutation c.603C > A (p.Asp201Glu) replaces aspartic acid with glutamic acid at codon 201 of the AGXT protein (p.Asp201Glu). This variant has been detected in some individuals and families affected with hyperoxaluria [9, 27, 30, 31]. This variant has been described to affect AGXT protein function, and it is considered as Pathogenic [20].

In contrast to the Arab Maghreb countries, which have the p.I244T mutation (Maghrebian mutation) as the most frequent mutation [12, 21], this mutation was detected in our study in only one patient as a heterozygous state combined with the new mutation (c.140G > A) at a frequency (1.1%).

The c.1078C > T (p.Arg360Trp) mutation was found in our cohort as a heterozygous state with c.584 T > G, it was associated with the Major allele. The Bioinformatics prediction program MutationTaster (www.mutationtaster.org), estimated the mutation c.1078C > T (p.Arg360Trp) to be disease causing [32]. We expect the mutation c.1078C > T (p.Arg360Trp) to be pathogenic as previously described [29].

The Exon 10 mutation c.997A > T (p.Arg333X) results in a premature termination codon (TGA) replacing a codon for amino acid 333 [33]. It was found in our cohort in a heterozygous state with c.508G > A mutations.

The mutation c.602A > T (p. Asp201Val) has not yet been defined in patients with primary hyperoxaluria type I. However, a mutation c.603C > A (p.Asp201Glu) at the same codon 201 leading to a different amino acid substitution has been found in patients with primary hyperoxaluria type I. furthermore the mutation c.602A > T (p. Asp201Val) is not annotated as single nucleotide polymorphism (SNP) in any database.

The new variant c.198C > A (p.Tyr66X) has not yet been previously described in patients with PH-1, while, a mutation c.198C > G (p.Tyr66X) at the same cDNA position already described [8]. The two mutations result in a premature termination codon (TAA) replacing a codon for amino acid 66.

In the other hand, the same finding was observed for the new variant c.140G > A (p.Gly47Glu) which has not yet been formerly defined in patients with PH-I. While, a mutation c.139G > A (p.Gly47Arg) at the same codon 47 was already known as pathogenic mutation [9]. The two mutations co-segregate with the Min allele, and the bioinformatics prediction program MutationTaster, estimated the mutation c.140G > A (p.Gly47Glu) to be disease causing.

In addition, the c.941C > T (p.Pro314Leu) mutation was reported with 1.1% of allele frequency in only one patient with heterozygous state. It was associated with the miner allele. This variation is a substitution in exon 9 for C to T at position 941, encoding a Pro to Leu substitution at residue 314. This variation was only reported in the ExAC database (http://exac.broadinstitute.org) as pathogenic mutation.

Finally, the, c.305 T > A (p.Val102Asp), mutation was identified in exon 2. It was founded in a homozygous state. In fact, the missense mutation (c.305 T > A) was not previously reported in any genome database. It was reported in this study for the first time, it was associated with the Miner allele. Analysis with MutationTaster predicted program estimated that the mutation was damaging mutations. On the other hand, the c.359-1_382del mutation was identified in exon 3. It was also founded in homozygous states. The (c.359-1_382del) mutation was previously described in Rumsby as an unpublished data [14].

The heterozygous only for one allele was detected in three patients. An AGXT MLPA was recommended in these patients in order to detect a huge insertion/deletion. In addition, complete sequencing of all known causative genes affecting glyoxylate metabolism should be considered.

### Limitation

The limitations of this study is that it is lacking in functional studies, which is necessary in order to be completely sure of the phenotypic effects of the new mutations.

In the other hand, some clinical information for some patients are unfortunately not available for us due to the current situation in Syria. Consequently, we were unable to present the full clinical information for some patient's as required for these kinds of studies.

## Conclusion

**Fig. 3:**
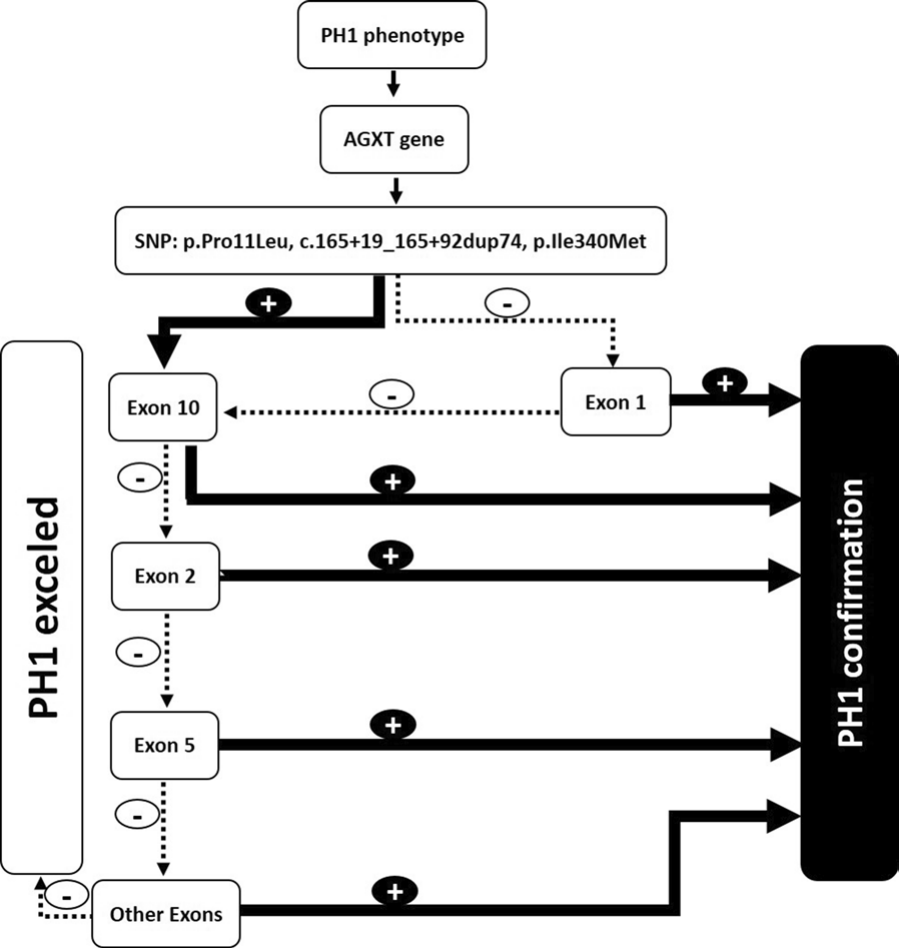
Decisional tree for molecular diagnosis of PH-1 in Syria. SNPs: single nucleotide polymorphisms

PH-1 is known as a clinically and genetically heterogeneous syndrome. We report here by our experience the AGXT gene mutation analysis in 45 unrelated probands with a definitive diagnosis of PH-1. DNA sequencing method was used for exon 1 essentially and then for exons 10, 2 and 5 of the AGXT gene. This technique can provide a useful, cost-effective process in Syrian PH-1 patients (Fig. 3). Molecular diagnosis of PH-1 in our country may be accomplished by following this process and would permit an accurate method for a precocious diagnosis in Syrian affected families (even without a clinical subtyping of PH) and detection of presymptomatic individuals. This will also avoid rapid progression to renal failure.

## Data Availability

The datasets generated and analysed during the current study for the Primary hyperoxaluria type 1 are available in the NCBI (accession number: (MIM 259900), https://www.ncbi.nlm.nih.gov/gene/?term=MIM%20259900). The web links of the relevant datasets were as follows: dbSNP (http://www.bioinfo.org.cn/relative/dbSNP%20Home%20Page.htm), ClinVar (https://www.ncbi.nlm.nih.gov/clinvar/), HGMD (http://www.hgmd.cf.ac.uk/ac/index.php), MutationTaster (www.mutationtaster.org), ExAC database (http://exac.broadinstitute.org).

## Abbreviations

PH-1: primary hyperoxaluria type 1
AGXT: alanine glyoxylate aminotransferase
Maj: major
Min: minor
